# ICD therapy in the elderly: a retrospective single-center analysis of mortality

**DOI:** 10.1007/s00399-021-00742-x

**Published:** 2021-01-29

**Authors:** Cornelia Scheurlen, Jan van den Bruck, Jonas Wörmann, Tobias Plenge, Arian Sultan, Daniel Steven, Jakob Lüker

**Affiliations:** 1 Department of Electrophysiology, Cologne, University Heart Center Cologne, Kerpener Str. 62, 50937 Köln, Germany; 2 Cardiology, Clinic Ernst von Bergmann, Potsdam, Germany

**Keywords:** Implantable cardioverter-defibrillator, Elderly patients, Mortality, Comorbidities, ICD therapies, Implantierbarer Cardioverter Defibrillator, Ältere Patienten, Sterblichkeit, Komorbiditäten, Schockabgaben

## Abstract

**Background:**

Current implantable cardioverter-defibrillator (ICD) guidelines do not impose age limitations for ICD implantation (IMPL) and generator exchange (GE); however, patients (pts) should be expected to survive for 1 year. With higher age, comorbidity and mortality due to non-sudden cardiac death increase. Thus, the benefit of ICD therapy in elderly pts remains unclear. Mortality after ICD IMPL or GE in pts ≥ 75 years was assessed.

**Methods:**

Consecutive pts aged ≥ 75 years with ICD IMPL or GE at the University Hospital Cologne, Germany, between 01/2013 and 12/2017 were included in this retrospective analysis.

**Results:**

Of 418 pts, 82 (20%) fulfilled the inclusion criteria; in 70 (55 = IMPL, 79%, 15 = GE, 21%) follow-up (FU) was available. The median FU was 3.1 years. During FU, 40 pts (57%) died (29/55 [53%] IMPL; 11/15 [73%] GE). Mean survival after surgery was 561 ± 462 days. The 1‑year mortality rate was 19/70 (27%) overall, 9/52 (17%) in pts ≥ 75 and 10/18 (56%) in pts ≥ 80 years. Deceased pts were more likely to suffer from chronic renal failure (85% vs. 53%, *p* = 0.004) and peripheral artery disease (18% vs. 0%, *p* = 0.02). During FU, seven pts experienced ICD shocks (four appropriate, three inappropriate). In primary prevention (*n* = 35) mortality was 46% and four pts experienced ICD therapies (two adequate); in secondary prevention (*n* = 35) mortality was 69% (*p* = 0.053) with three ICD therapies (two adequate).

**Conclusion:**

Mortality in ICD pts aged ≥ 80 years was 56% at 1 and 72% at 2 years in this retrospective analysis. The decision to implant an ICD in elderly pts should be made carefully and individually.

## Abstract (de)

**Hintergrund:**

Die aktuellen Leitlinien zur Verwendung von implantierbaren Kardioverter-Defibrillatoren (ICD) geben keine Altersgrenze für ICD-Implantation (IMPL) und Aggregatwechsel (AW) vor, Patienten (Pat.) sollten aber eine Lebenserwartung von einem Jahr haben. Mit steigendem Alter nehmen Komorbiditäten und die Mortalität aufgrund eines nicht-plötzlichen Todes zu. Der Nutzen der ICD-Therapie für ältere Pat. ist daher unklar. In dieser Arbeit sollte die Mortalität nach ICD-IMPL oder AW bei Pat. ≥ 75 Jahre untersucht werden.

**Methodik:**

Konsekutive Pat. ≥ 75 Jahre, die zwischen 01/2013 und 12/2017 an der Uniklinik Köln einer ICD-IMPL oder einem AW unterzogen wurden, wurden in diese retrospektive Analyse eingeschlossen.

**Ergebnisse:**

Von 418 Pat. erfüllten 82 (20 %) die Einschlusskriterien, bei 70 (55 = IMPL, 79 %; 15 = AW, 21 %) lagen Follow-up(FU)-Daten vor. Die mediane FU-Zeit betrug 3,1 Jahre. Während des FU starben 40 (57 %) Pat. (29/55 [53 %] IMPL; 11/15 [73 %] AW). Die mittlere Überlebenszeit nach Eingriff lag bei 561 ± 462 Tagen. Die 1‑Jahres-Mortalität betrug in der Gesamtgruppe 19/70 (27 %), bei 75- bis 79-Jährigen 9/52 (17 %) und bei ≥ 80-Jährigen 10/18 (56 %). Verstorbene Pat. litten häufiger an einer chronischen Niereninsuffizienz (85 % vs. 53 %, *p* = 0,004) und peripheren arteriellen Verschlusskrankheit (18 % vs. 0 %, *p* = 0,02). Während des FU kam es bei 7 Patienten zu ICD-Schockabgaben (4 adäquat, 3 inadäquat). In der primärprophylaktischen Gruppe (*n* = 35) lag die Mortalität bei 46 %, bei 4 Pat. kam es zu ICD-Schockabgaben (2 adäquat). In der sekundärprophylaktischen Gruppe (*n* = 35) betrug die Mortalität 69 % (*p* = 0,053), ICD-Schockabgaben waren bei 3 Pat. zu verzeichnen (2 adäquat).

**Schlussfolgerung:**

Die Mortalität von Pat. ≥ 80 Jahre mit ICD lag in dieser retrospektiven Analyse bei 56 % (1 Jahr) bzw. 72 % (2 Jahre). Die Entscheidung über eine ICD-IMPL sollte bei Älteren sorgfältig und individuell abgewogen werden.

ICD implantation is an effective treatment for life-threatening ventricular tachyarrhythmias. There are no specific guidelines on the approach to elderly patients with an ICD indication.

This article presents a retrospective single-center analysis to assess mortality in elderly patients after ICD implantation.

## Introduction

Implantable cardioverter-defibrillators (ICD) are an effective treatment for life-threatening ventricular tachyarrhythmias. As a therapy to prevent sudden cardiac death, ICD have been widely adopted in recent decades. ICD are used both for secondary prevention in patients (pts) with documented ventricular arrhythmia, as well as for primary prevention in pts with reduced left ventricular ejection fraction (EF) < 35%.

In Germany, more than 25,000 implantations (IMPL) of ICD and more than 10,000 generator exchanges (GE) were performed in 2017. Of those new implantations, 12% were in pts aged 80 years or older [11]. In comparison with other European countries like Sweden and Switzerland (Table 1), Germany has significantly higher figures regarding both the number of ICD implantations per 1 million citizens and the share of implantations for pts aged 80 years or above [11].

**Table 1:** Implantable cardioverter-defibrillator (ICD) implantations in 2017 [11]

ICD implantations in 2017	Germany	Sweden	Switzerland
ICD implantations per 1 million citizens	*312*	139	131
Share of pts 80 years or above	*12.3%*	4.0%	2.9%

The current ICD guidelines do not impose age limitations on ICD IMPL and GE. However, pts should have a predicted life expectancy of at least 1 year to be considered for ICD implantation [15].

The predicted life expectancy is of particular relevance in elderly pts with a guideline indication for ICD therapy, given their age, burden of comorbidities and their risk for potential complications during or after ICD implantation [20, 22]. Furthermore, the probability of sudden cardiac death compared to non-sudden death decreases with increasing age [10].

Thus far, elderly and old pts are underrepresented in randomized ICD trials, both for primary prevention [1, 8, 9, 12] and secondary prevention [3, 23]. The mean age in randomized ICD trials is < 70 years [1, 3, 8, 9, 12, 23] and, therefore, results may not be applicable in elderly pts. In clinical practice, the decision for or against ICD implantation in elderly pts is often made case-by-case, depending on known comorbidities. In an aging population, the number of elderly pts with an indication for ICD IMPL or GE will continue to increase. Hence, data regarding the benefit of ICD therapy in the elderly is needed.

The present study aims to assess mortality after ICD IMPL or GE in elderly pts in a retrospective single-center analysis. We included pts aged 75 and older that were implanted with a transvenous ICD or that had received an ICD GE.

## Methods

### Study population

All pts 75 years or older undergoing ICD IMPL or GE between January 2013 and December 2017 at the University Hospital of Cologne, Germany, were considered for this retrospective analysis. Inclusion criteria were age 75 years or older, transvenous ICD IMPL or GE. All pts gave written informed consent to the procedure. Pts aged < 75 years, as well as patients that had undergone pacemaker surgery or subcutaneous ICD implantation, were excluded.

Patient subgroups were defined based on age: aged 75–79 (75–79) vs. ≥ 80 years (80+). Furthermore, primary (PRIM) vs. secondary prevention (SEC) indication were compared.

### Data collection and clinical follow-up

The following data were systematically collected for all pts: personal data, comorbidities, medication, medical history, as well as device and procedure characteristics.

Follow-up was conducted by phone, assessment of available medical records and by contacting the respective general practitioner. During follow-up, data on all-cause mortality as well as ICD therapies (shocks and antitachycardia pacing) were collected.

### Study endpoints

The primary endpoint was defined as all-cause mortality during follow-up. Secondary endpoints were occurrence of ICD therapies during follow-up and rate of comorbidities.

### Statistical analysis

Statistical analysis was performed. A *p*-value < 0.05 was considered statistically significant. Baseline and procedural data were shown as mean and standard deviation, median for continuous variables and counts and percentages for categorical variables. Statistical significance was evaluated by t‑test for normally distributed continuous variables and by chi-test for categorical variables.

## Results

### Study population

**Fig. 1:**
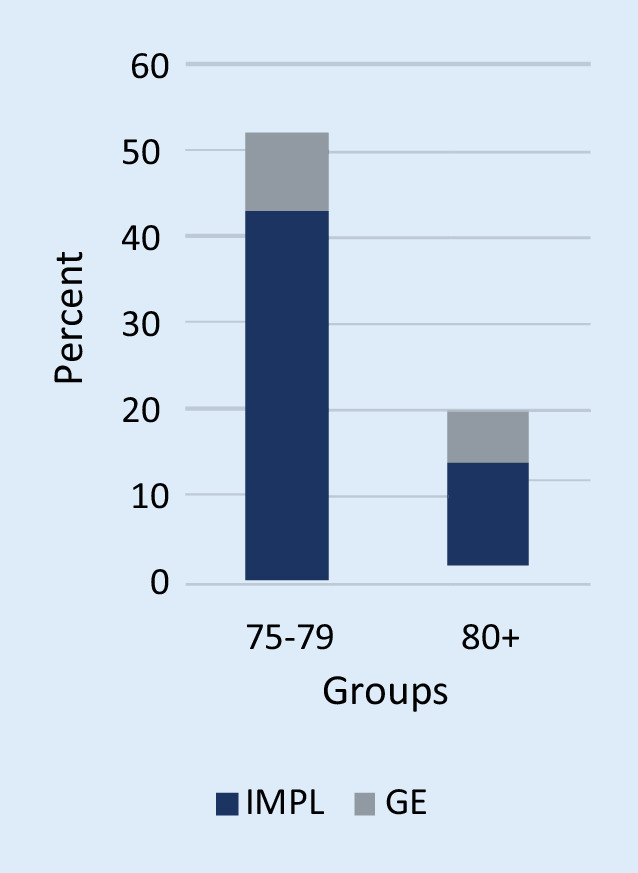
Implantation (*IMPL*) vs. generator exchange (*GE*) in the two observed groups—group 75–79 and group 80+—in percent

**Fig. 2:**
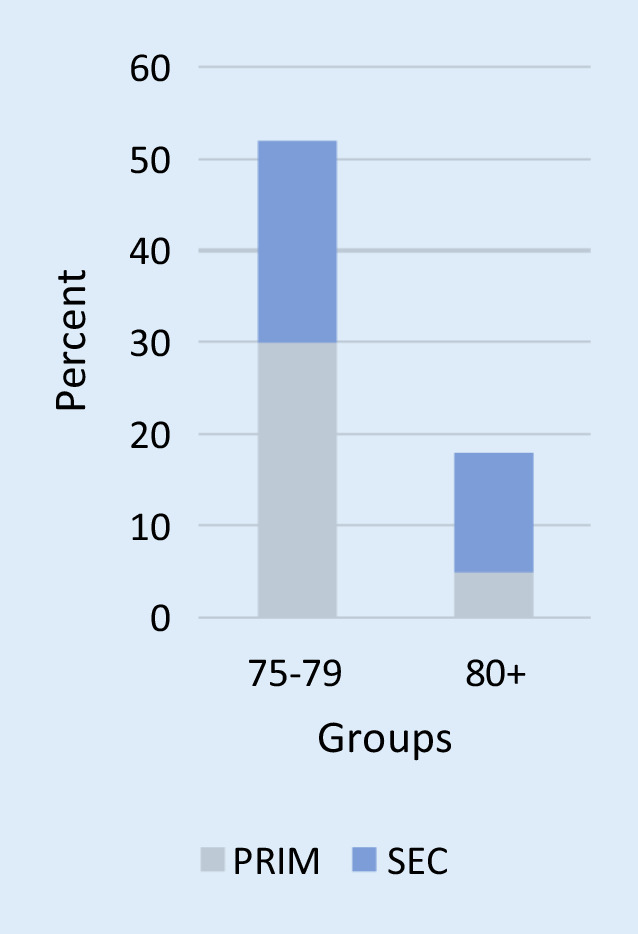
Primary (*PRIM*) vs. secondary (*SEC*) prevention indication in the two observed groups—group 75–79 and group 80+—in percent

**Fig. 3:**
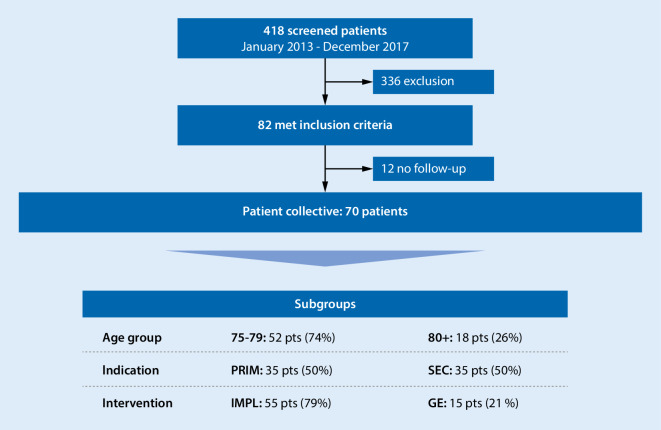
Flowchart of study design. *pts* patients, *PRIM* primary prevention, *SEC* secondary prevention, *IMPL* implantation, *GE* generator exchange

Of the 418 screened pts, 82 (20%) fulfilled the inclusion criteria. In 12 pts (15%), no follow-up information was available; the remaining 70 pts were included in the assessment—thereof 52 in group 75–79 (74%) and 18 in group 80+ (26%). The median age was 78.6 ± 3.7 years (group 75–79: 76.8 ± 1.4 years, group 80+: 83.7 ± 3.8 years), 87% male (group 75–79: 87%, group 80+: 89%). In 55 pts (79%), an ICD IMPL was performed (75–79: 83%, 80+: 67%), in 15 pts (21%) a GE (Fig. 1). The ICD indication was primary prevention in 35 pts (50%), 75–79: 30 (58%), 80+: 5 (28%) (Fig. 2). Figure 3 presents an overview of the different observed groups. The mean follow-up time was 3.3 years (75–79: 3.4 years, 80+: 3.1 years). In the 80+ group, more pts had received an ICD for secondary prevention (72%) compared to the 75–79 group, *p* = 0.03. Beside that and age, there were no statistically significant differences between the groups regarding baseline characteristics (Table 2).

**Table 2:** Baseline characteristics

Baseline characteristics	All patients	Group 75–79	Group 80+	*p*-Value
Pts, *n (%)*	70 (100%)	52 (74%)	18 (26%)	0.80
Age, *average, (SD)*	78.6 (3.7)	76.8 (1.4)	83.7 (3.8)	–
Male, *n (%)*	61 (87%)	45 (87%)	16 (89%)	0.80
ICD implantation, *n (%)*	55 (79%)	43 (83%)	12 (67%)	0.15
Generator exchange, *n (%)*	15 (21%)	9 (17%)	6 (33%)	
Primary prevention, *n (%)*	35 (50%)	30 (58%)	5 (28%)	0.03^a^
Secondary prevention *n (%)*	35 (50%)	22 (42%)	13 (72%)	

Continuous data is summarized as means ± standard deviation. Categorical data is presented as number (percent). Two observed groups: 75–79 and 80+

^a^Statistically significant p‑value

*pts* Patients, *n* number, *SD* standard deviation, *ICD* implantable cardioverter-defibrillator

### Mortality

After ICD IMPL or GE, 40/70 pts (57%) died during the follow-up period of 3.3 years. Mortality was significantly higher in group 80+ (16 of 18, 89%) compared to group 75–79 (24 of 52, 46%) (*p* = 0.002). The 1‑year and 2‑year mortality after the procedure was 27% and 39%, respectively. In the group of pts aged 80+, it was significantly higher at 72% and 56%, respectively, compared with the group of ages 75–79 with 27% and 17% (*p* < 0.001 and *p* = 0.002), respectively. Table 3 illustrates the mortality in the two observed groups.

**Table 3:** Mortality in the two observed groups: 75–79 and 80+

Mortality	All patients	Group 75–79	Group 80+	*p*-Value
During follow-up period, *n (%)*	40 (57%)	24 (46%)	16 (89%)	0.002^*a*^
1‑Year mortality, *n (%)*	19 (27%)	9 (17%)	10 (56%)	0.002^*a*^
2‑Year mortality, *n (%)*	27 (39%)	14 (27%)	13 (72%)	<0.001^*a*^

^*a*^Statistically significant *p*-value; *n* number

**Fig. 4:**
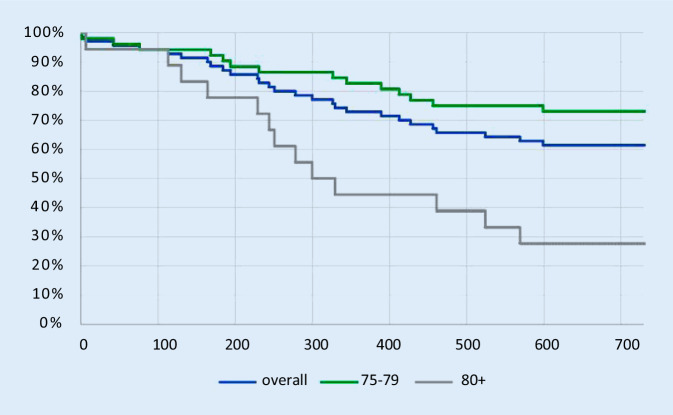
Kaplan-Meier curve illustrating overall survival and survival for each group (age 75–79 and age 80+) in days after implantable cardioverter-defibrillator implantation or generator exchange

The average survival after ICD intervention of the deceased pts was 1.5 ± 1.3 years for the entire cohort and 1.8 ± 1.4 years and 1.2 ± 1.0 years in the group 75–79 and 80+, respectively. This difference was not statistically significant (*p* = 0.09). Figure 4 illustrates mortality during follow-up.

### Comorbidities

The laboratory findings pre-implantation and clinical history were analysed. Besides a significantly higher number of strokes in group 75–79 (*p* = 0.04), there were no statistically significant differences in comorbidities between these two groups. Chronic renal failure was defined as glomerular filtration rate (GFR) < 60 ml/min [5]. Table 4 shows comorbidities in each group, 75–79 and 80+.

**Table 4:** Comorbidities in the two observed groups: 75–79 and 80+

Comorbidities	All patients	Group 75–79	Group 80+	*p*-Value
Pts *n* (%)	70 (100%)	52 (100%)	18 (100%)	
CAD, *n* (%)	59 (84%)	44 (85%)	15 (83%)	0.90
CABG, *n* (%)	28 (40%)	20 (38%)	8 (44%)	0.66
DCM, *n* (%)	3 (4%)	1 (2%)	2 (11%)	0.10
LVEF < 35%, *n* (%)	42 (60%)	32 (62%)	10 (56%)	0.66
AH, *n* (%)	50 (71%)	39 (75%)	11 (61%)	0.26
DM Type II, *n* (%)	24 (34%)	20 (38%)	4 (22%)	0.21
AF, *n* (%)	35 (50%)	26 (50%)	9 (50%)	1.00
COPD, *n* (%)	4 (6%)	2 (4%)	2 (11%)	0.25
Stroke, *n* (%)	10 (14%)	10 (19%)	0 (0%)	0.04^*a*^
PAD, *n* (%)	7 (10%)	6 (12%)	1 (6%)	0.47
Chronic renal insufficiency GFR < 60, *n* (%)	50 (71%)	37 (71%)	13 (72%)	0.93
Terminal renal failure, *n* (%)	3 (4%)	2 (4%)	1 (6%)	0.76

^a^Statistically significant *p*-value

*Pts* Patients, *n* number, *CAD* coronary artery disease, *CABG* coronary artery bypass graft, *DCM* dilatative cardiomyopathy, *LVEF* left ventricular ejection fraction, *AH* arterial hypertension, *DM* diabetes mellitus, *AF* atrial fibrillation, *COPD* chronic obstructive pulmonary disease, *PAD* peripheral artery disease, *GFR* glomerular filtration rate

Table 5 compares the presence of comorbidities in deceased vs. alive pts. This comparison shows that among the deceased pts, more pts suffered from chronic renal failure (deceased: 34 pts, 85%, vs. alive: 16 pts, 53%, *p* = 0.004). Also, significantly more of the deceased pts had peripheral artery disease (PAD) (seven pts, 18%), compared with surviving pts (0 pts, 0%) (*p* = 0.02). All other observed comorbidities showed no statistically significant differences between deceased and the surviving pts.

**Table 5:** Comorbidities in deceased and alive pts (number and percent of all pts with this comorbidity)

Comorbidities	All patients	Deceased	Surviving	*p*-Value
Pts, *n* (%)	70 (100%)	40 (57%)	30 (43%)	
CAD, *n* (%)	59 (84%)	34 (85%)	25 (83%)	0.85
CABG, *n* (%)	28 (40%)	18 (45%)	10 (33%)	0.32
DCM, *n* (%)	3 (4%)	2 (5%)	1 (3%)	0.73
LVEF < 35%, *n* (%)	42 (60%)	23 (58%)	19 (63%)	0.62
AH, *n* (%)	50 (71%)	29 (73%)	21 (70%)	0.82
DM Type II, *n* (%)	24 (34%)	12 (30%)	12 (40%)	0.38
AF, *n* (%)	35 (50%)	19 (48%)	16 (53%)	0.63
COPD, *n* (%)	4 (6%)	4 (10%)	0 (0%)	0.07
Stroke, *n* (%)	10 (14%)	6 (15%)	4 (13%)	0.84
PAD, *n* (%)	7 (10%)	7 (18%)	0 (0%)	0.02^*a*^
Chronic renal failure GFR < 60, *n* (%)	50 (71%)	34 (85%)	16 (53%)	0.004^*a*^
Terminal renal failure	3 (4%)	3 (8%)	0 (0%)	0.13

^*a*^Statistically significant p‑value

*Pts* patients, *n* number, *CAD* coronary artery disease, *CABG* coronary artery bypass graft, *DCM* dilatative cardiomyopathy, *EF* left ventricular ejection fraction, *AH* arterial hypertension, *DM* diabetes mellitus, *AF* atrial fibrillation, *COPD* chronic obstructive pulmonary disease, *PAD* peripheral artery disease, *GFR* glomerular filtration rate

### Primary vs. secondary prevention indication

Regarding the underlying ICD indication, the full analysis set (70 pts) was evenly split into two groups of primary (35 pts, 50%) and secondary (35 pts, 50%) prevention indication. Of the 35 pts in the primary prevention group, 30 pts were 75–79 years old (86%) and five pts 80 years and older (14%). Of the 35 pts in the secondary prevention group, 22 pts were 75–79 years old (63%) and 13 pts 80 years and older (37%).

Table 6 illustrates the share of deceased vs. surviving pts per age group, who had their ICD implanted for primary vs. secondary prevention. A strong trend towards higher mortality among pts with an ICD for secondary prevention (24 of 35 deceased, 69%) compared to those with ICD for primary prevention (16 of 35 deceased, 46%) was observed (*p* = 0.053). Among the pts with an ICD for secondary prevention, all of the pts in the age group 80+ (13 pts) and half of the pts in the age group 75–79 (11 pts) died during the post-operative period.

**Table 6:** Primary and secondary prevention indication and deceased pts in primary and secondary prevention indication in the two observed groups: 75–79 and 80+

Primary vs. secondary prevention	Age group	Deceased	Surviving	Total
Primary prevention	75–79	13 (43%)	17 (57%)	30
	80+	3 (60%)	2 (40%)	5
	*Total*	*16 (46%)*	*19 (54%)*	*35*
Secondary prevention	75–79	11 (50%)	11 (50%)	22
	80+	13 (100%)	(0%)	13
	*Total*	*24 (69%)*	*11 (31%)*	*35*

### Adequate and inadequate ICD therapies

During the follow-up of 3.3 ± 1.2 years, seven pts experienced ICD therapies: four adequate for ventricular tachycardia or ventricular fibrillation, and 3 inadequate, none of which were directly related to a mortality event. Four shocks occurred in pts 75–79, three in group 80+, four in primary and three in the secondary prevention indication group (Table 6).

One of the four pts with adequate ICD therapy died during the follow-up period, while the other three pts were alive at the end of the follow-up period.

## Discussion

### Main findings

The main findings of this retrospective analysis are, firstly, that more than half of the pts aged 80 years or older included in this study did not survive for at least 1 year after ICD implantation. Secondly, the deceased pts were more likely to suffer from chronic kidney disease and peripheral artery disease. And thirdly, the rate of adequate shocks was low in this cohort of elderly and old pts.

#### Mortality

The limited additional benefit from ICD intervention in elderly pts found in this study might appear contradictory to several large-scale randomized ICD trials, which demonstrated a reduction in mortality by means of ICD implantation in primary as well as in secondary prevention in selected patient groups [1, 3, 8, 9, 12, 23]. However, the mean age in these randomized ICD trials was < 70 years [1, 3, 8, 9, 12, 23]. Therefore, while not questioning the general benefits of ICD intervention for prevention in the average population, the present analysis offers a complementary perspective, specifically on elderly pts among whom the benefit of ICD intervention appears to be limited. A meta-analysis by Santangeli et al. of the above-mentioned trials already demonstrated a smaller survival benefit of prophylactic ICD implantation for the subset of elderly pts compared to younger pts [18]. Another meta-analysis of those trials found that ICD therapy in elderly pts (≥75 years old) reduced neither all-cause mortality nor sudden cardiac death [7].

According to current ICD guidelines, there must be a life expectancy of at least 1 year to be considered for ICD implantation [15]. Given that, in this retrospective analysis, more than half of the pts in the age group 80+ did not reach the 1‑year survival after ICD intervention.

This conclusion on declining incremental benefit of ICD intervention for the elderly is in line with results of Goonewardene et al., who found in their retrospective study that > 40% of 80+ pts died within a mean follow up period of 3 years after implantation [6]. Similarly, in a study by Zakine et al., 36% of the pts with primary prevention indication aged 80 years or older died after a mean follow up of 3 years [22]. The findings from Krahn et al., who highlight the decreasing probability of sudden cardiac death compared to non-sudden death with increasing age, further emphasise the call for the conservative indication of ICD interventions for elderly pts [10].

More scrutiny before deciding to implement an ICD in elderly pts seems to be appropriate—in particular in the presence of certain co-morbidities.

#### Comorbidities

The present study found a number of co-morbidities, in particular chronic renal disease and peripheral artery disease, to be significantly more often prevalent among the pts who deceased during the follow-up period compared with survivors. This implies that both kidney disease and peripheral artery disease may serve as predictors for limited life expectancy after ICD intervention. Given the higher comorbidity burden in elderly pts [13, 20] in general, and therefore also in elderly pts with ICD indication, this further skews the mortality rate after ICD intervention towards elderly.

The observed correlation between chronic kidney disease and increased mortality after ICD intervention is in line with a meta-analysis of three large randomized trials by Pun et al. In that analysis it was shown that chronic kidney disease (GFR < 60 ml/min) actually reverses the survival benefit after ICD implantation in pts with primary prevention indication [16].

Barsheshet et al. developed a risk score, consisting of five risk factors, to evaluate a long-term benefit of ICD implantation in MADIT-II pts: Besides age (>70) and the risk factors New York Heart Association functional class II, QRS duration (>0.12 s) and atrial fibrillation, this score factors blood urea nitrogen (>26 mg/dl) as a risk indicator [2]. The study also highlights that in high-risk pts, who do not benefit from ICD therapy, statistically significant higher creatinine levels are observed.

In their prospective registry, Yung et al. also identified multiple predictors of mortality after ICD interventions—among others peripheral artery disease in PRIM and SEC prevention indication as well as chronic kidney disease only in prophylactic indication [21].

In light of those findings, the benefit of ICD intervention in elderly pts seems particularly questionable in the presence of chronic renal disease or peripheral artery disease as co-morbidities.

#### Adequate and inadequate ICD therapies

A low rate of ICD shocks was observed in this cohort. Several studies showed a similar shock rate in younger and elderly pts [6, 17, 21, 22]. However, Van Rees et al. found that, while the shock rate was similar between different age groups, the expected life prolongation from ICD therapy was significantly less for elderly compared to younger pts [17]. Yung et al. also describe an increase in all-cause mortality with higher age without a decline in shock rates.

Regarding the observed inadequate shocks, it is to be assumed that they impaired quality of life, caused psychological distress and in themselves carried the risk of increasing the all-cause mortality of the respective pts [4, 14, 19].

In summary, this retrospective single-center analysis questions the benefit of a broad application of ICD therapy in pts aged 80 years and older, in particular in the presence of chronic kidney disease and peripheral artery disease as co-morbidities. These findings are well in line with previous studies. While not questioning the benefit of ICD intervention for the average population, this study indicates its limitations in elderly pts. In light of these findings, ICD indication in older pts should be assessed carefully with a special focus on relevant comorbidities taken into account in shared decision-making.

### Limitations

This study is a single-center study and retrospective in nature. The number of pts, and in particular the number of pts in the elderly group (80+), was relatively small (18 of 70 pts). The follow-up was assessed partially by phone and without comprehensive ICD interrogations—therefore some ICD shocks (adequate and inadequate) may not have been captured in the data collection.

## Conclusion

Short-term mortality in pts aged ≥ 75 is high after ICD IMPL and ICD GE. In this analysis, the 1‑year and 2‑year mortality after IMPL or GE were 56% and 72%, respectively, for pts aged 80 and older. Chronic kidney disease and peripheral artery disease were mortality-relevant comorbidities. The rate of adequate ICD therapy was low. Randomised trials are needed to further evaluate the benefits and risks of ICD therapy for elderly pts.

## Practical conclusion

- Elderly and old pts with an ICD IMPL or GE indication according to guideline recommendations should be carefully evaluated.
- The current ICD guidelines do not impose age limitations on ICD IMPL and GE. However, pts should have a predicted life expectancy of at least 1 year to be considered for ICD implantation.
- ICD intervention in elderly pts seems particularly questionable in the presence of severe comorbidities, e.g. chronic renal disease or peripheral artery disease as co-morbidities.

## References

[1] Bardy GH, Lee KL, Mark DB, Poole JE, Packer DL, Boineau R, Domanski M, Troutman C, Anderson J, Johnson G, McNulty SE, Clapp-Channing N, Davidson-Ray LD, Fraulo ES, Fishbein DP, Luceri RM, Ip JH (2005). Amiodarone or an implantable cardioverter—defibrillator for congestive heart failure. N Engl J Med.

[2] Barsheshet A, Moss AJ, Huang DT, McNitt S, Zareba W, Goldenberg I (2012). Applicability of a risk score for prediction of the long-term (8-year) benefit of the Implantable cardioverter-defibrillator. J Am Coll Cardiol.

[3] Connolly SJ, Gent M, Roberts RS, Dorian P, Roy D, Sheldon RS, Mitchell LB, Green MS, Klein GJ, O’Brien B (2000). Canadian Implantable Defibrillator Study (CIDS)—A randomized trial of the implantable cardioverter defibrillator against amiodarone. Circulation.

[4] Daubert JP, Zareba W, Cannom DS, McNitt S, Rosero SZ, Wang P, Schuger C, Steinberg JS, Higgins SL, Wilber DJ, Klein H, Andrews ML, Hall WJ, Moss AJ (2008). Inappropriate implantable cardioverterDefibrillator shocks in MADIT II—frequency, mechanisms, predictors, and survival impact. J Am Coll Cardiol.

[5] Eknoya G, Lameire N, Eckardt KU (2013). KDIGO 2012 clinical practice guideline for the evaluation and management of chronic kidney disease. Kidney Int.

[6] Goonewardene M, Barra S, Heck P, Begley D, Fynn S, Virdee M, Grace A, Agarwal S (2015). Cardioverter-defibrillator implantation and generator replacement in the octogenarian. Europace.

[7] Healey JS, Hallstrom A, Kuck KH, Nair G, Schron EP, Roberts RS, Morillo CA, Connolly SJ (2007). Role of the implantable defibrillator among elderly patients with a history of life-threatening ventricular arrhythmias. Eur Heart J.

[8] Kadish A, Dyer A, Daubert JP, Quigg R, Estes M, Anderson KP, Calkins H, Hoch D, Goldberger J, Shalaby A, Sanders WE, Schaechter A, Levine JH (2004). Prophylactic defibrillator implantation in pts with nonischemic dilated cardiomyopathy. N Engl J Med.

[9] Køber L, Thune JJ, Nielsen JC, Haarbo J, Videbæk L, Korup E, Jensen G, Hildebrandt P, Steffensen FH, Bruun NE, Eiskjær H, Brandes A, Thøgersen AM, Gustafsson F, Egstrup K, Videbæk R, Hassager C, Svendsen JH, Høfsten DE, Torp-Pedersen C, Pehrson S (2016). Defibrillator implantation in pts with nonischemic systolic heart failure. N Engl J Med.

[10] Krahn AD, Connolly SJ, Roberts RS, Gent M (2004). Diminishing proportional risk of sudden death with advancing age: implications for prevention of sudden death. Am Heart J.

[11] Markewitz A (2019). Jahresbericht 2017 des Deutschen Herzschrittmacherund Defibrillator-Registers – Teil 2: Implantierbare Kardioverter-Defibrillatoren (ICD). Herzschr Elektrophys.

[12] Moss AJ, Zareba W, Hall WJ, Klein H, Wilber DJ, Cannom DS, Daubert JP, Higgins SL, Brown MW, Andrews AL (2002). Prophylactic implantation of a defibrillator in pts with myocardial infarction and reduced ejection fraction. N Engl J Med.

[13] Murad K, Goff DC, Morgan TM, Burke GL, Bartz TM, Kizer JR, Chaudhry SI, Gottdiener JS, Kitzmann DW (2015). Burden of comorbidities and functional and cognitive impairments in elderly patients at the initial diagnosis of heart failure and their impact on total mortality—the cardiovascular health study. J Am Coll Cardiol.

[14] Poole JE, Johnson GW, Hellkamp AS, Anderson J, Callans DJ, Raitt MH, Reddy RK, Marchlinski FE, Yee R, Guarnieri T, Talajic M, Wilber DJ, Fishbein DP, Packer DL, Mark DB, Lee KL, Bardy GH (2008). Prognostic importance of defibrillator shocks in pts with heart failure. N Engl J Med.

[15] Priori SG, Blomström-Lundqvist C, Mazzanti A, Blom N, Borggrefe M, Camm J, Elliott PA, Fitzsimons D, Hatala R, Hindricks G, Kirchhof P, Kjeldsen K, Kuck KH, Hernandez-Madrid A, Nikolaou N, Norekval TM, Spaulding C, Van Veldhuisen DJ (2015). 2015 ESC Guidelines for the management of pts with ventricular arrhythmias and the prevention of sudden cardiac death. Eur Heart J.

[16] Pun HP, Al-Khatib SM, Han JY, Edwards R, Bardy GH, Bigger JT, Buxton AE, Moss AJ, Lee KL, Steinman R, Dorian P, Hallstrom A, Cappato R, Kadish AH, Kudenchuk PJ, Mark DB, Hess PL, Inoue LYT, Sanders GD (2014). Implantable cardioverter defibrillators for primary prevention of sudden cardiac death in CKD: a meta-analysis of patient-level—data from 3 randomized trials. Am J Kidney Dis.

[17] van Rees J, Borleffs JW, Thijssen J, de Bie M, van Erven L, Cannegieter SC, Bax JJ, Schalij MJ (2012). Prophylactic implantable cardioverter-defibrillator treatment in the elderly: therapy, adverse events and survival gain. Europace.

[18] Santangeli P, Di Biase L, Dello Russo A, Casella M, Bartoletti S, Santarelli P, Pelargonio G, Natale A (2010). Meta-analysis: age and effectiveness of prophylactic implantable cardioverter-defibrillators. Ann Intern Med.

[19] Schron EB, Exner DV, Yao Q, Jenkins LS, Steinberg JS, Cook JR, Kutalek SP, Friedman PL, Bubien RS, Page RL, Powell J (2002). Quality of life in the antiarrhythmics versus implantable defibrillators trial—impact of therapy and influence of adverse symptoms and defibrillator shocks. Circulation.

[20] Wolff JL, Starfield B, Anderson G (2002). Prevalence, expenditures, and complications of multiple chronic conditions in the elderly. Arch Intern Med.

[21] Yung D, Birnie D, Dorian P, Healey JS, Simpson CS, Crystal E, Krahn AD, Khaykin Y, Cameron D, Chen Z, Lee DS (2013). Survival after implantable cardioverter-defibrillator implantation in the elderly. Circulation.

[22] Zakine C, Garcia R, Narayanan K, Gandjbakhch E, Algalarrondo V, Lellouche N, Perier MC, Fauchier L, Gras D, Bordachar P, Piot O, Babuty D, Sadoul N, Defaye P, Deharo JC, Klug D, Leclercq C, Extramiana F, Boveda S, Marijon E (2019). Prophylactic implantable cardioverter-defibrillator in the very elderly. Europace.

[23] Zipes DP, Wyse DG, Friedman PL, Epstein AE, Hallstrom AP, Greene HL, Schron EB, Domanski M (1997). A Comparison of antiarrhythmic-drug therapy with implantable defibrillators in pts resuscitated from near-fatal ventricular arrhythmias. N Engl J Med.

